# Maternal Prenatal Factors and Child Adiposity in Associations with Cardiometabolic Risk Factors in Term-Born Chinese Children at the Age of 2 Years

**DOI:** 10.3390/nu15153342

**Published:** 2023-07-27

**Authors:** Fengxiu Ouyang, Jonathan C. Wells, Guang-Hui Zhang, Kun Du, Xia Wang, Lixiao Shen, Zhong-Cheng Luo, Jun Zhang

**Affiliations:** 1 Ministry of Education and Shanghai Key Laboratory of Children’s Environmental Health, Xinhua Hospital, Shanghai Jiao Tong University School of Medicine, Shanghai 200092, China; 2 Childhood Nutrition Research Centre, Population, Policy and Practice Research Department, Great Ormond Street Institute of Child Health, University College London, London WC1N 1EH, UK; 3 Department of Clinical Laboratory Test, Xinhua Hospital, Shanghai Jiao Tong University School of Medicine, Shanghai 200092, China; 4 Department of Obstetrics and Gynecology, Prosserman Centre for Population Health Research, Lunenfeld-Tanenbaum Research Institute, Mount Sinai Hospital, Institute of Health Policy, Management and Evaluation, University of Toronto, Toronto, ON M5T 3M6, Canada

**Keywords:** maternal prenatal factors, infancy, adiposity, anthropometric measures, blood pressure, cardiometabolic risk factors, the first 1000 days

## Abstract

Early growth has long-lasting associations with adult metabolic health. However, the association of adiposity with cardiometabolic risk factors in toddlers remains poorly understood. This study aimed to examine the association of maternal prenatal factors and child adiposity with child cardiometabolic risk factors among boys and girls aged 2 years. This was a birth cohort study of 549 term-born children in Shanghai, China, with follow-up data at the age of 2-years. Child anthropometric and adiposity measurements included weight, length, and skinfold thickness (triceps, subscapular, and abdominal). Child cardiometabolic risk factors included random morning plasma glucose, serum insulin, lipids, and systolic and diastolic blood pressure (SBP, DBP). At 2 years, overweight/obesity (weight-for-length z score, ZWFL > 2) was associated with 12.6 (95%CI 7.7, 17.4) mmHg higher SBP, and 7.9 (4.1, 11.8) mmHg higher DBP in boys, with similar results observed in girls. Maternal hypertensive disorders of pregnancy were associated with 3.0 (0.1, 5.8) higher SBP, 3.17 (0.90, 5.44) mmHg higher DBP, 0.24 (0.01,0.47) mmol/L higher plasma glucose, and 0.26 (0.01,0.51) mmol/L higher serum triglycerides after adjustment for child age, sex, and ZWFL. Maternal hypertensive disorders of pregnancy and child overweight/obesity were associated with higher SBP and DBP at the age of 2 years.

## 1. Introduction

The prevalence of non-communicable diseases (NCDs) is increasing globally, and cardiovascular disease (CVD) is the leading cause of premature death worldwide [1,2]. NCDs are responsible for 74% of global deaths [2], of which more than 80% occur in low- and middle-income countries (LMICs) [3]. These major NCDs are CVD, diabetes, cancer, and chronic respiratory diseases [2]. Metabolic risk factors include elevated blood pressure, overweight and obesity (OWO), hyperglycemia, and hyperlipidemia [2]. Elevated blood pressure is a key risk factor of CVD [1], and hypertension is more prevalent in LMICs than in high-income countries (HICs) [1,4]. In HICs, CVD mortality has declined for decades, mediated by lower mean SBP and DBP blood pressure [1,3].

Growth during the first 1000 days (from conception to 2 years of age) has long-lasting associations with cardiometabolic health in later life [5,6]. The first 1000 days of life provide a unique window of opportunity to shape a healthy life trajectory and prevent NCDs [7]. OWO are major risk factors for NCDs in both adults and adolescents [8]. However, data are scarce concerning the association of adiposity with cardiometabolic risk factors in toddlers [9,10]. Infant weight gain and high infant body mass index (BMI) might be associated with an increased risk of later cardiometabolic disorders [11]. 

The prevalence of overweight and obesity, defined using BMI, has been increasing worldwide over the past four decades [12], while the prevalence of child undernutrition has been decreasing. In China, the prevalence of overweight was 6.8% and that of obesity was 3.6% in children under the age of 6 years during 2015–2019 [12]. There are sex differences in the prevalence of overweight and obesity in Chinese children [13]; boys have higher overweight/obesity prevalence than girls [13]. It is known that body composition differs between adult men and women [14], whereby men on average have greater lean mass, while women have more fat mass as a proportion of body weight [14,15]. Body composition also differs by sex in prepubertal children, school-age children, and adolescents [12,13,14], and already at birth, boys have longer length and higher lean mass than girls [16]. 

In this study, we aimed to examine the associations of maternal prenatal factors and child growth and adiposity measures with metabolic risk factors (blood glucose/insulin, serum lipids, and blood pressure) among boys and girls aged 2 years. A secondary aim was to evaluate sex differences in body composition at the age of 2 years. 

## 2. Methods

### 2.1. Study Population

This study used data from children at the age of 2 years from the Shanghai Obesity and Allergy Cohort Study, a prospective study which was conducted in Shanghai, China. The primary objective of this prospective cohort study was to evaluate early life environmental and maternal risk factors for childhood obesity and allergic diseases [17]. Pregnant women were enrolled when they were admitted to two study hospitals for delivery, namely, Xinhua Hospital and the International Peace Maternity and Child Hospital, two large tertiary teaching hospitals, from June 2012 to February 2013. Pregnant women were enrolled in this study if they (1) were having routine prenatal care at one of the study hospitals, (2) had a singleton pregnancy, (3) planned to reside in Shanghai in the next 2 years, and (4) were willing to participate in the study and provided written informed consent. A total of 1243 women were eligible and enrolled. The trained study nurses conducted face-to-face questionnaire-based interviews to collect general demographic information, social-economic status, health behavior habits, prepregnancy weight, and pregnancy-related information. The women usually delivered their babies in the 1–2 days following our questionnaire interview. After delivery, the study nurses reviewed the mothers’ medical records and abstracted data on prenatal care, laboratory reports, pregnancy complications, labor and delivery course, gestational age at birth, infant sex, birth weight, and length, before the mother was discharged from hospital. Among 1243 women, 829 children had at least one postnatal follow-up visit. There were 23 children born preterm (gestational age < 37 weeks), and they were excluded from this report. Among the remaining 806 infants, 549 completed follow-up visits at the age of 2 years.

During postnatal follow-up at 6 months of age, a web-based questionnaire was used to collect infant data. The infant diet mode from birth to months (formula feeding, exclusive breastfeeding, and mixed breastfeeding) was obtained based on self-report at the age of six months from the parents. At ages 1 and 2 years, children were invited for postnatal follow-up visits at Xinhua Hospital to assess infant growth, feeding mode, and passive smoking information. The 2 years postnatal follow-up was conducted from June 2014 to April 2015. At this follow-up, study staff conducted a face-to-face questionnaire interview; a nurse measured length and weight; two pediatricians measured MUAC, skinfold thickness, and blood pressure; and infant morning blood samples were collected from the child for assays of metabolic health biomarkers. This report used cross-sectional data of 549 term-born children at the age of 2 years. We obtained written informed consent from all participants. The study was approved by the institutional review board of Xinhua Hospital; Approval No: XHEC-C-2012-002; Approval Date: 21 May 2012.

### 2.2. Hypertensive Disorders in Pregnancy and Gestational Diabetes Mellitus

Hypertensive disorders in pregnancy and information on gestational diabetes mellitus were abstracted from medical records. Hypertensive disorders in pregnancy were diagnosed by obstetricians per clinical guidelines. GDM was defined using the recommendations of the International Association of Diabetes and Pregnancy Study Groups (IADPSG) [18]. Specifically, a 75 g oral glucose tolerance test (OGTT) was applied at 24–28 weeks of gestation. GDM was diagnosed if any of the following plasma glucose values were reached: (1) fasting plasma glucose: ≥5.1 mmol/L; (2) 1 h: ≥10.0 mmol/L; and (3) 2 h: ≥8.5 mmol/L [18,19].

### 2.3. Anthropometric Measurements in Children at the Age of 2 Years

Weight was measured to the nearest 100 g (Seca 956 Scale) with the children in light underclothes. Length was measured to the nearest 0.1 cm in a supine position (Seca 416 Scale). Head circumference was measured to the nearest 0.1 cm with inelastic tape. Mid-upper arm circumference was measured to the nearest 0.1 cm with inelastic tapes. Anthropometric measures followed WHO protocols (https://www.who.int/toolkits/child-growth-standards; accessed on 26 May 2023).

Skinfold thicknesses were measured at the triceps, subscapular, and abdominal sites to the nearest 0.5 mm by two study pediatricians using Lange calipers (Beta Technology, Santa Cruz, CA, USA). With 70–90% of the total adipose tissue located subcutaneously, skinfold thicknesses reflect the total body fat [20].

Sex-specific z-scores of length-for-age (LAZ), weight-for-length (ZWFL), weight-for-age (WAZ), BMI-for-age (BMIZ) were calculated using the WHO Child Growth Standards (WHO 2006) (https://www.who.int/toolkits/child-growth-standards; accessed on 1 March 2023) [19]. Overweight was defined as ZWFL > 2, Obesity as ZWFL > 3, wasting as ZWFL < −2, stunting as LAZ < −2 SD, and underweight as WAZ < −2 SD.

### 2.4. Blood Pressures at the Age of 2 Years

BP was measured using a mercury sphygmomanometer using an appropriate size cuff for arm circumference, at least thirty minutes after the child arrived. Three readings were taken 1 min apart and the average was used in the analysis.

### 2.5. Assessment of Blood Glucose, Insulin and Lipids at the Age of 2 Years

Random morning plasma glucose, serum insulin, and lipids (triglyceride (TG); total cholesterol (TC); high-density lipoprotein (HDL); low-density lipoprotein (LDL)) were measured in the laboratory of Xinhua Hospital, a tertiary hospital lab certified by the China National Accreditation Service for Conformity Assessment. Serum TG, TC, HDL, LDL, and plasma glucose were measured using a Hitachi 917 auto-analyzer, and serum insulin was measured on a Beckman DXI 800 autoanalyzer. Parents were asked to record the breakfast time of their child.

### 2.6. Statistical Analysis

We presented the maternal and child characteristics according to infant sex. We used χ^2^ tests for categorical data and Student’s *t*-tests for continuous data to test for differences between the two groups (Table 1). We also presented the mean (SD) of child anthropometric adiposity measures and other cardiometabolic factors by infant sex (Table 1). Natural logarithm transformation of insulin was used in all comparisons.

To examine the cross-sectional relationships of child growth and adiposity measures with cardiometabolic risk factors in children, age-adjusted partial Pearson’s correlation coefficients were computed in boys and girls. We also calculated age-adjusted partial Pearson’s correlation coefficients among cardiometabolic factors in boys and girls at the age of 2 years.

Linear regression models were used to examine associations of ZWFL (<−1, −1 to 1, >1 to 2, >2) with each cardiometabolic factor with adjustment for child age in boys and girls, and to examine the association of hypertensive disorders of pregnancy and GDM with cardiometabolic factors with adjustment for child age, sex and ZWFL as a continuous variable. All analyses were performed using SAS 9.2 software (SAS Institute, Cary, NC, USA). 

## 3. Results

Of the 549 term-born infants, 51.9% were boys. The mean gestational age was 39.0 weeks in both boys and girls. Birth weight was greater in boys (3505 ± 403 g) than in girls (3370 ± 426 g; *p* < 0.001). Thirteen (4.6%) boys and nine (3.5%) girls were overweight or obese at the age of 2 years (Table 1). Five boys (1.8%) were stunted, one (0.4%) boy and one (0.4%) girl were wasted, and none of the children were underweight at the age of 2 years. Overall, 3.1% and 0.9% of infants were overweight and obese, 0.5% of infants wasted, and 1.0% stunted, respectively.

### 3.1. The Anthropometric Measures and Metabolic Risk Factors in Boys and Girls at the Age of 2 Years

As shown in Table 1, at the age of 2 years, boys had greater weights (13.1 ± 1.4 vs. 12.5 ± 1.5 kg; *p* < 0.001), lengths (89.5 ± 3.1 vs. 88.1 ± 3.1 cm; *p* < 0.001), head circumferences (49.0 ± 1.3 vs. 47.9 ± 1.3 cm; *p* < 0.001) and BMIs (16.4 ± 1.4 vs. 16.2 ± 1.4 kg/m^2^; *p* = 0.02) than girls. In contrast, girls had larger summed skinfold thicknesses than boys (23.5 ± 5.3 vs. 22.3 ± 4.8 mm; *p* = 0.009). As expected, there were no sex differences in LAZ, WAZ, ZWFL, or BMIZ. 

Boys had higher plasma glucose (5.02 ± 0.56 vs. 4.87 ± 0.61; *p* = 0.01) and lower serum LDL (2.31 ± 0.49 vs. 2.41 ± 0.56; *p* = 0.04) than girls. No differences were observed in serum insulin, TC, triglycerides, HDL, SBP, or DBP between boys and girls. 

### 3.2. Child Adiposity Measures and Cardiometabolic Risk Factors at the Age of 2 Years

**Figure 1:**
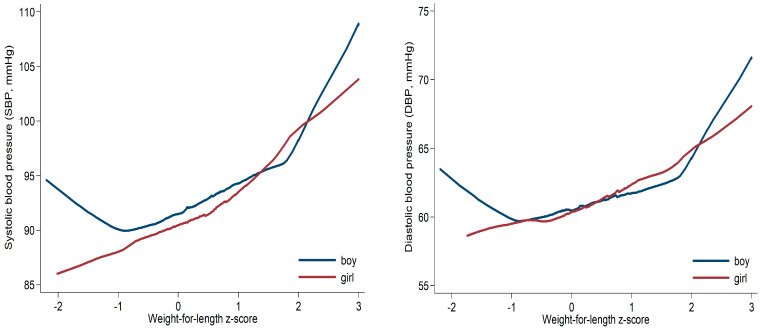
Lowess regression plot of systolic (SBP) and diastolic (DBP) blood pressures by weight-for-length z-score in boys and girls at age 2 years.

As shown in Table 2 and Figure 1, the anthropometric growth and adiposity measures (ZWFL, BMIZ, WAZ, MUAC, and skinfold thickness) were all positively correlated with SBP and DBP in both boys and girls. The age-adjusted partial correlation coefficient (r) ranged 0.27–0.55 (all *p* < 0.01) between anthropometric measures and BP in boys and ranged 0.26–0.53 (all *p* < 0.01) in girls. As for length measures (LAZ), its correlation with SBP was 0.23–0.26 in boys and girls, and 0.21 with DBP in girls, but there was no association with DBP in boys (r = 0.08, *p* > 0.05).

No correlations were observed between anthropometric measures and other metabolic risk factors in girls. In boys, no correlations were observed between anthropometric measures and serum lipid profiles (TG, HDL, LDL, and TC) or plasma glucose, except that positive associations were observed between serum insulin (in natural logarithm transform) and weight parameters: WAZ (r = 0.17, *p* < 0.05), ZWFL (r = 0.17, *p* < 0.05), BMI (r = 0.15, *p* < 0.05) and BMIZ (r = 0.17, *p* < 0.05)) in boys (Table 2). 

### 3.3. Child OWO and Cardiometabolic Risk Factors at the Age of 2 Years

As child OWO is assessed using ZWFL using WHO criteria, we further explored its association with SBP and DBP (Table 3, Figure 1) as well as plasma glucose, serum insulin, triglycerides, TC, LDL and HDL concentrations (Table S1). As shown in Table 3 and Figure 1, a 1SD increase in ZWFL, SBP increased by 3.2 (95% CI 2.2, 4.2) mmHg in boys and 3.0 (2.0, 4.1) mmHg in girls, and DBP increased by 1.7 (0.9, 2.5) mmHg in both boys and girls. Of note, compared to children with normal weight, child OWO was associated with 12.6 (7.7, 17.4) mmHg higher SBP and 7.9 (4.1, 11.8) mmHg higher DBP in boys, and 12.5 (6.9, 18.2) mmHg higher SBP and 7.0 (2.5, 11.4) mmHg higher DBP in girls, respectively.

As for other metabolic risk factors, no associations were observed of child OWO with plasma glucose, triglyceride, TC, LDL, and HDL concentrations in both boys and girls at the age of 2 years. ZWFL was associated with 0.19 (95% CI 0.07, 0.31) higher serum insulin concentration (in logarithm transform, pmol/L) in boys, suggesting higher insulin resistance with greater ZWFL. No association was observed in girls. 

Regarding correlations between cardiometabolic factors (Table S2), as expected, SBP was correlated with DBP (r = 0.69 in boys, 0.61 in girls), plasma glucose was correlated with serum insulin (r = 0.43 in boys, 0.26 in girls), serum HDL was positively correlated with serum TC and negatively correlated with serum TG, and LDL was correlated with HDL and highly correlated with TC (r = 0.91 in boys, 0.94 in girls).

### 3.4. Maternal Hypertensive Disorders in Pregnancy and GDM in Associations with Cardiometabolic Risk Factors in Term-Born Children at the Age of 2 Years

As shown in Table 4, maternal hypertensive disorders of pregnancy were associated with 3.0 mm Hg higher SBP (95% CI 0.1, 5.8 mm Hg), 3.2 (0.9, 5.4) mmHg higher DBP, 0.24 (0.01, 0.47) mmol/L higher plasma glucose, and 0.26 (0.01,0.51) mmol/L higher serum triglycerides, with adjustment for child age, sex, and ZWFL. As shown in Table 5, maternal GDM was associated with 2.2 (0.1, 4.3) mm Hg higher SBP and 0.16 (−0.01, 0.33) mmol/L higher plasma glucose. No associations were observed between GDM and other cardio- metabolic risk factors in children at the age of 2 years (Table 5).

## 4. Discussion

Child anthropometric adiposity measures were positively correlated with SBP and DBP in both boys and girls at the age of 2 years. Weight measures (ZWFL and BMIZ) were associated with higher serum insulin levels in boys, but not in girls. Maternal hypertensive disorders of pregnancy were associated with higher offspring SBP and DBP, and maternal GDM was associated with higher offspring SBP at the age of 2 years. This study confirmed sex differences in adiposity measures in children aged 2 years. There was no association between child anthropometric adiposity measures and serum lipid profiles (TG, HDL, LDL, and TC) or plasma glucose in this study. 

The association of adiposity measures and child OWO with cardiometabolic risk factors among children at the age of 2 years has not been assessed previously. We observed positive associations between adiposity and blood pressure (SBP and DBP) among children at the age of two years, consistent with adiposity and blood pressure associations among older children and adolescents (age 8–18 years) [20,21]. A previous 4-year follow-up cohort study in adolescents showed that a decrease in BMI from 13 to 17 years was associated with lower SBP and a lower risk of hypertension at 17 years [22]. Higher BMI was also found to be associated with higher SBP and DBP at the ages of 4 and 7 years [23]. A pooled data analysis of five birth cohorts in LMICs indicated that body size was the strongest influential factor related to BP, and BP was affected by faster weight gain at any age [24]. Not surprisingly, child adiposity measures were highly correlated with higher SBP and DBP at the age of 2 years in this study. 

Higher birth weight was associated with higher child weight measures (for example, BMIZ or ZWFL), and SGA was associated with lower child weight measure at ages of 2, 4, and 7 years [17,23]. However, birth weight has been negatively associated with SBP at the age of 4 years and lower SBP and DBP at the age of 7 years [23]. Another study among children with OWO (mean age 11.8 years) also reported that, compared to being born appropriate for gestational age (AGA), SGA was associated with a higher risk of hypertensive BP and low HDL, while large for gestational age (LGA) was associated with a lower risk of hypertensive BP [25]. Our recent study likewise showed that SGA was associated with higher SBP, and LGA with lower DBP at two years of age in boys, though not in girls, adjusting for child current ZWFL [17]. This can be explained by the capacity-load mode, where birth weight is a marker of capacity and childhood BMI or skinfolds are markers of load [6]. 

There are few data for comparison regarding the association of infant adiposity with metabolic risk factors (blood glucose/insulin and serum lipids) in young children. The associations of adiposity measures (or OWO) with cardiometabolic outcomes varied by age. Past studies on aspects of impaired fasting glucose, HOMA-IR, and insulin resistance were mostly focused on older children aged 2–19 years [26,27]. While children aged nine to fourteen years with metabolic syndrome (MS) had a higher BMI, percent body fat, and skinfold thickness compared to those without MS, more than 50% of those with MS had normal weight without OWO [28]. Regarding blood glucose and insulin, our study found that ZWFL and WAZ were positively correlated with log (serum insulin) in boys, but not girls, at 2 years. No associations were found between birth weight and other cardiometabolic factors (fasting serum glucose, TG, and LDL-C) [23]. 

Serum lipid levels are related to age, child sex, and maturation [29], and they remain similar during childhood, but change during puberty [29]. A previous study reported positive associations of BMI with TG, TC, and LDL-C among children aged 9–11 years in Iran [30]. That study also reported that TG, TC, and LDL-C concentrations were higher in children with OWO than in normal weight children [30]. In another study of children aged 7.5–13 years in China, children with OWO showed higher TG, LDL-C, insulin levels, and lower HDL-C than normal weight children [31]. We did not find any association of child adiposity measures with blood glucose or serum lipid profiles (TG, HDL, LDL, and TC) at the age of 2 years in this study. The difference in findings might be due to the younger age in this study. 

A previous study reported that maternal hypertensive disorders were associated with offspring higher BP (both SBP and DBP) at the age of 7 years and throughout childhood and adolescence [32]. The results of our paper confirmed this at younger years. Only a few studies have examined the mechanism underlying this association [33,34]. A recent study suggested that the association between maternal and offspring BP might be explained in part by shared family-based genetic and environmental factors, instead of a causal relationship by “in utero” exposure [34]. Higher BP in offspring of mothers with hypertensive disorders during pregnancy may reflect genetic susceptibility to develop high BP [35]. The exact underlying mechanism of this association is still uncertain [33].

This study observed no association between maternal preeclampsia and child BP. This may be due to the small sample size of preeclampsia cases, which might limit the statistical power of this study. Most of the previous studies have also observed no consistent association between maternal preeclampsia and child BP in older age [34]. 

In fact, hypertensive disorders during pregnancy may have critical potential consequences for mothers, not only for children. Preeclampsia is one of the leading causes of maternal and neonatal morbidity and mortality [36]. In addition, hypertensive disorders during pregnancy were associated with an increased risk of cardiovascular diseases (including myocardial infarction, heart failure, hypertension, and/or stroke) for mothers in later life [37]. The early identification of women at risk for hypertensive disorders and early intervention may promote both maternal and child immediate and future health.

We observed sex differences in body composition among young children at the age of 2 years. Body composition (fat mass and fat-free mass) changes with age from birth throughout infancy to adulthood [38,39]. In a small study of healthy full-term infants, girls had a greater percent fat but lower fat-free mass than boys at 1 and 3 months of age; however, by 6 months of age, the sex difference disappeared [40]. Results were consistent in children aged 0–6 months but inconsistent by age > 6 months in previous studies. A recent study showed that weight, length, head circumference, and skinfold thickness (triceps and subscapular) predicted fat mass well through infancy (the coefficient of determination, R2, ranged between 0.77 and 0.87 at the age of 3 days, 3 months, 13 months) [41]. In our study, girls had higher skinfold thicknesses, but lower weight, length, BMI, and head circumference than boys at the age of 2 years. Our findings are biologically plausible. Sex differences in body composition can be explained in part by sex hormones [42,43]. Ovaries support follicle growth, and estradiol content of the ovaries peaks during the first 6 months of life in girls [43,44]. Fat accumulation is relatively fast during the first 6 months [45]. A previous study reported that sex differences in body composition emerged in the first 5–6 months of life, with lower fat accumulation in males [42,43]. Such sex difference might be due to endogenous testosterone production in male infants [42,43]. In addition, a previous study reported that boys appear to present more “impaired” fasting glucose than girls, while girls had higher insulin resistance in obese children aged 2–19 years [27]. The results in this study suggested a difference in general at the age of 2 years.

This prospective study has its strengths. We collected high-quality clinical data on multiple pre-, peri-, and postnatal risk factors for NCDs and a wide range of cardiovascular outcomes (serum lipids, blood pressure, etc.) in toddlers, an understudied age group. This study also has some limitations. This report only examined children up to 2 years, and long-term follow-up of these children will allow us to understand the long-term health impact of child adiposity. Also, because it is challenging to collect fasting blood samples from children at this young age, the random morning blood samples were collected to measure plasma glucose, serum insulin, and lipid levels. Thus, caution is needed when interpreting plasma glucose and serum triglyceride levels of children in this study, which might be affected by fasting/breakfast status.

In this study, pregnant women were enrolled from 2012 to 2013, and postnatal 2-year follow-up was conducted from 2014 to 2015 in Shanghai, China. In the past 10 years (with the exception of the COVID-19 pandemic period), there was little change in terms of maternal and child health care (or maternal and child health) that could potentially affect the results if this study was repeated today in Shanghai. The results of this study should be able to reflect today’s situation in Shanghai, China. However, caution is needed when generalizing our findings to other populations. 

## 5. Conclusions

In this study, we found the following: (1) overweight/obesity was associated with higher blood pressure as early as in infancy; (2) maternal hypertensive disorders of pregnancy were associated with higher SBP and DBP in term-born Chinese children; (3) we confirmed sex difference in adiposity measures in young children. Boys had greater length, weight, and BMI than girls, while girls had greater skinfold thickness than boys at the age of 2 years. Further independent cohort studies are needed to confirm these findings. The findings of this study underscore the importance of the first 1000 days as a potential window in early prevention of CVD.

## Figures and Tables

**Table 1:** Maternal and child characteristics and metabolic risk factors in 549 children aged 2 years.

	Boys (*n* = 285)	Girls (*n* = 264)	*p* Value
Mothers			
Maternal prepregnancy BMI (kg/m^2^) categories			
<18.5	47 (16.5)	41 (15.5)	0.73
18.5–23.9	185 (65.1)	180 (68.2)	
24–27.9	42 (14.8)	29 (11.0)	
>28	10 (3.5)	14 (5.3)	
Gestational Weight Gain			
Adequate	108 (38.3)	83 (31.6)	0.48
Excessive	124 (44.0)	140 (53.2)	
Inadequate	50 (17.7)	40 (15.2)	
GDM, yes	36 (12.6)	33 (12.5)	0.96
Hypertensive disorders in pregnancy			
none	258 (90.5)	252 (95.5)	0.01
Chronic hypertension/gestational hypertention	19 (6.7)	11 (4.2)	
Preeclampsia	8 (2.8)	1 (0.4)	
Children			
Age, months	23.8 ± 0.6	23.8 ± 0.5	0.92
Birth weight (gram)	3504.8 ± 403.1	3369.6 ± 425.9	<0.001
Gestational age (weeks)	39.0 ± 1.0	39.0 ± 1.0	0.55
Anthropometric measures			
Weight, kg	13.1 ± 1.4	12.5 ± 1.5	<0.001
Length, cm	89.5 ± 3.1	88.1 ± 3.1	<0.001
Head circumference, cm	49.0 ± 1.3	47.9 ± 1.3	<0.001
BMI, kg/m^2^	16.4 ± 1.4	16.2 ± 1.4	0.02
MUAC, cm	16.0 ± 1.2	15.8 ± 1.2	0.14
Skinfold thickness, mm			
Triceps	9.1 ± 1.8	9.4 ± 2.1	0.03
Subscapular	6.6 ± 1.7	6.9 ± 1.8	0.04
Abdominal	6.7 ± 1.9	7.2 ± 2.0	0.007
Sum of three sites	22.3 ± 4.8	23.5 ± 5.3	0.009
Weight-for-length z-score	0.44 ± 0.99	0.40 ± 0.92	0.65
Weight-for-age z-score	0.65 ± 0.92	0.65 ± 0.93	0.98
Length-for-age z-score	0.61 ± 1.01	0.59 ± 0.95	0.74
BMI-for-age z-score	0.41 ± 1.01	0.43 ± 0.93	0.82
Head circumference-for-age z-score	0.57 ± 0.93	0.56 ± 0.91	0.94
Arm circumference-for-age z-score	0.64 ± 0.98	0.72 ± 0.91	0.38
Subscapular skinfold-for-age z-score	0.29 ± 1.30	0.34 ± 1.20	0.59
Triceps skinfold-for-age z-score	0.70 ± 0.94	0.76 ± 1.04	0.48
Weight-for-length z-score	n (%)		
<−2 (wasting)	1 (0.4)	1 (0.4)	0.91
−2 to 2 (normal)	271 (95.1)	253 (96.2)	
>2 to 3 (overweight)	10 (3.5)	7 (2.7)	
>3 (obesity)	3 (1.1)	2 (0.8)	
Length-for-age z-score < −2 (stunting)	5 (1.8)	0 (0.0)	0.06
Cardiometabolic risk factors			
Glucose, mmol/L	5.02 ± 0.56	4.87 ± 0.61	0.01
Insulin, pmol/L	37.80 ± 33.91	38.90 ± 32.01	0.73
log(insulin), pmol/L	3.26 ± 0.92	3.35 ± 0.84	0.28
TC, mmol/L	4.12 ± 0.69	4.22 ± 0.74	0.16
Triglycerides, mmol/L	1.11 ± 0.68	1.06 ± 0.55	0.40
HDL, mmol/L	1.41 ± 0.35	1.37 ± 0.29	0.18
LDL, mmol/L	2.31 ± 0.49	2.41 ± 0.56	0.04
SBP, mm Hg	93.12 ± 8.17	91.88 ± 7.60	0.10
DBP, mm Hg	61.35 ± 6.24	61.29 ± 5.72	0.92

DBP: diastolic blood pressure; HDL: high-density lipoprotein; SBP: systolic blood pressure; LDL: low-density lipoprotein; TC: total cholesterol; and MUAC: Mid-upper arm circumference). *t*-test for continuous variables. Fisher’s exact test for categorical variables.

**Table 2:** Age-adjusted partial Pearson correlation coefficients (r) between child anthropometric measures and metabolic risk factors in term-born Chinese boys and girls at the age of 2 years.

Child Anthropometric Measures	Child Cardiometabolic Risk Factors							
	SBP	DBP	Glucose	log(insulin)	TC	TG	HDL	LDL
Boys								
Weight (kg)	0.49 ***	0.29 ***	−0.08	0.16 *	0.03	0.001	0.11	−0.03
Length (cm)	0.23 **	0.08	−0.15	0.07	−0.06	0.03	0.08	−0.08
BMI (kg/m^2^)	0.43 ***	0.30 ***	0.02	0.15 *	0.08	−0.02	0.07	0.03
Length-for-age z-score	0.24 **	0.08	−0.15	0.07	−0.06	0.03	0.08	−0.08
Weight-for-age z-score	0.47 ***	0.27 ***	−0.08	0.17 *	0.04	0.01	0.10	−0.02
Weight-for-length z-score	0.43 ***	0.28 ***	−0.0004	0.17 *	0.09	−0.004	0.08	0.04
BMI-for-age z-score	0.40 ***	0.27 ***	0.02	0.16 *	0.11	−0.01	0.07	0.05
MUAC (cm)	0.55 ***	0.39 ***	−0.03	0.05	0.05	−0.004	0.05	0.03
Sum of skinfold thickness at three sites (mm)	0.46 ***	0.31 ***	−0.03	0.02	0.13	0.05	−0.03	0.08
Girls								
Weight (kg)	0.44 ***	0.32 ***	0.04	0.15	−0.01	−0.02	0.14	−0.02
Length (cm)	0.26 ***	0.21 **	0.09	0.13	−0.09	−0.04	0.10	−0.07
BMI (kg/m^2^)	0.40 ***	0.28 ***	−0.03	0.10	0.06	0.003	0.11	0.05
Length-for-age z-score	0.26 **	0.21 **	0.09	0.13	−0.09	−0.04	0.10	−0.07
Weight-for-age z-score	0.43 ***	0.30 ***	0.04	0.14	−0.003	−0.03	0.13	−0.005
Weight-for-length z-score	0.41 ***	0.28 ***	−0.03	0.10	0.06	−0.01	0.12	0.04
BMI-for-age z-score	0.39 ***	0.26 ***	−0.04	0.09	0.07	−0.002	0.11	0.05
MUAC (cm)	0.46 ***	0.39 ***	0.01	0.05	−0.01	−0.01	0.05	−0.02
Sum of skinfold thickness at three sites (mm)	0.53 ***	0.44 ***	0.02	−0.04	0.10	−0.05	0.13	0.06

* *p* < 0.05; ** *p* < 0.01; *** *p* < 0.001. Sample size varies from 558 to 424 in serum measures due to missing blood samples. MUAC: Mid-upper arm circumference.

**Table 3:** The association between child overweight and obesity (OWO) and blood pressures at the age of 2 years.

Child Adiposity Measures		Boys				Girls		
	n	Mean ± SD	β (95% CI)	*p* Value		Mean ± SD	β (95% CI)	*p* Value
ZWFL	SBP (mmHg)							
−1 to 1	153	91.6 ± 6.6	Ref.		137	90.9 ± 6.5	Ref.	
>1 to 2	47	96.1 ± 8.4	4.3 (1.8, 6.8)	0.0008	47	95.0 ± 8.1	4.0 (1.7, 6.3)	0.0005
>2 (OWO)	10	104.4 ± 15.7	12.6 (7.7, 17.4)	<0.0001	6	103.3 ± 9.8	12.5 (6.9, 18.2)	<0.0001
<−1	17	91.6 ± 7.4	0.07 (−3.7, 3.8)	0.97	16	86.4 ± 7.5	−4.2 (−7.8, −0.7)	0.02
ZWFL continuous			3.2 (2.2, 4.2)	<0.0001			3.0 (2.0, 4.1)	<0.0001
	DBP (mmHg)							
−1 to 1	153	60.5 ± 5.9	Ref.		137	60.6 ± 5.7	Ref.	
>1 to 2	47	62.7 ± 5.6	2.1 (0.1, 4.1)	0.04	47	63.1 ± 5.0	2.4 (0.6, 4.2)	0.009
>2 (OWO)	10	68.5 ± 10.3	7.9 (4.1, 11.8)	<0.0001	6	67.5 ± 8.2	7.0 (2.5, 11.4)	0.002
<−1	17	61.2 ± 5.5	0.7 (−2.3, 3.7)	0.65	16	59.3 ± 4.6	−1.1 (−4.0, 1.8)	0.46
ZWFL continuous			1.7 (0.9, 2.5)	<0.0001			1.8 (1.0, 2.6)	<0.0001

ZWFL: weight-for-length z-score; All models were adjusted for child age.

**Table 4:** The associations between maternal hypertensive disorders in pregnancy and cardiometabolic risk factors in term-born children at the age of 2 years.

	Child Cardiometabolic Risk Factors at Age 2 Years			
Maternal Prenatal Factors	n	Mean ± SD	β (95% CI)	*p* Value
Hypertensive disorders in pregnancy		SBP, mm Hg		
none	402	92.3 ± 7.75	Ref.	
Chronic hypertension/gestational hypertention	26	96.69 ± 9.61	2.95 (0.08, 5.82)	0.04
Preeclampsia	5	90 ± 7.07	−3.63 (−9.99, 2.74)	0.26
Hypertensive disorders in pregnancy		DBP, mm Hg		
none	400	61.1 ± 5.7	Ref.	
Chronic hypertension/gestational hypertention	26	65 ± 8.83	3.17 (0.90, 5.44)	0.006
Preeclampsia	5	60 ± 6.12	−1.46 (−6.50, 3.57)	0.57
Hypertensive disorders in pregnancy		Glucose, mmol/L		
none	384	4.93 ± 0.59	Ref.	
Chronic hypertension/gestational hypertention	26	5.18 ± 0.43	0.24 (0.01, 0.47)	0.04
Preeclampsia	7	5.23 ± 0.82	0.25 (−0.18, 0.69)	0.26
Hypertensive disorders in pregnancy		log (insulin), pmol/L		
none	383	3.29 ± 0.88	Ref.	
Chronic hypertension/gestational hypertention	26	3.62 ± 0.97	0.31 (−0.04, 0.65)	0.08
Preeclampsia	7	3.16 ± 0.8	−0.16 (−0.81, 0.49)	0.63
Hypertensive disorders in pregnancy		TC, mmol/L		
none	384	4.17 ± 0.73	Ref.	
Chronic hypertension/gestational hypertention	26	4.09 ± 0.64	−0.08 (−0.37, 0.20)	0.56
Preeclampsia	7	4.26 ± 0.33	0.09 (−0.44, 0.63)	0.74
Hypertensive disorders in pregnancy		Triglycerides, mmol/L		
none	384	1.07 ± 0.59	Ref.	
Chronic hypertension/gestational hypertention	26	1.34 ± 0.95	0.26 (0.01, 0.51)	0.04
Preeclampsia	7	0.81 ± 0.45	−0.3 (−0.76, 0.17)	0.21
Hypertensive disorders in pregnancy		HDL, mmol/L		
none	384	1.39 ± 0.32	Ref.	
Chronic hypertension/gestational hypertention	26	1.34 ± 0.38	−0.06 (−0.19, 0.07)	0.38
Preeclampsia	7	1.74 ± 0.29	0.33 (0.09, 0.57)	0.007
Hypertensive disorders in pregnancy		LDL, mmol/L		
none	384	2.37 ± 0.54	Ref.	
Chronic hypertension/gestational hypertention	26	2.26 ± 0.43	−0.1 (−0.31, 0.11)	0.35
Preeclampsia	7	2.34 ± 0.22	0.02 (−0.38, 0.41)	0.93

All models were adjusted for child age, sex, and weight-for-length z-score (ZWFL).

**Table 5:** The associations between gestational diabetes mellitus (GDM) and cardiometabolic risk factors in term-born children at the age of 2 years.

Maternal Prenatal Factors	Child Cardiometabolic Risk Factors at Age 2 Years			
	N	Mean ± SD	β (95% CI)	*p* Value
GDM		SBP, mm Hg		
no	382	92.34 ± 7.7	Ref.	
yes	51	93.96 ± 9.37	2.2 (0.10, 4.30)	0.04
		DBP, mm Hg		
no	380	61.18 ± 5.72	Ref.	
yes	51	62.41 ± 7.7	1.6 (−0.07, 3.28)	0.06
		Glucose, mmol/L		
no	364	4.92 ± 0.58	Ref.	
yes	53	5.09 ± 0.61	0.16 (−0.01, 0.33)	0.06
		log (insulin), pmol/L		
no	363	3.29 ± 0.87	Ref.	
yes	53	3.41 ± 0.93	0.13 (−0.12, 0.38)	0.31
		TC, mmol/L		
no	364	4.17 ± 0.71	Ref.	
yes	53	4.13 ± 0.77	−0.04 (−0.24, 0.17)	0.73
		Triglycerides, mmol/L		
no	364	1.10 ± 0.64	Ref.	
yes	53	0.93 ± 0.45	−0.17 (−0.35, 0.00)	0.06
		HDL, mmol/L		
no	364	1.4 ± 0.31	Ref.	
yes	53	1.36 ± 0.39	−0.04 (−0.13, 0.06)	0.45
		LDL, mmol/L		
no	364	2.36 ± 0.53	Ref.	
yes	53	2.33 ± 0.53	−0.03 (−0.18, 0.13)	0.74

All models were adjusted for child age, sex, and weight-for-length z-score (ZWFL) at the age of 2 years.

## Data Availability

Data is not readily available because access to the deidentified participant research data must be approved by the research ethics board on a case-by-case basis. Please contact the corresponding author Dr. F Ouyang for assistance in data access request.

## Supplementary Materials

The following supporting information can be downloaded at: https://www.mdpi.com/article/10.3390/nu15153342/s1, Table S1: The associations between child overweight and obesity and cardio-metabolic risk factors. Table S2: Age-adjusted partial Pearson correlation coefficients between cardio-metabolic risk factors among term-born Chinese boys and girls aged 2 years.
