# Supplementary material for: Maternal Prenatal Factors and Child Adiposity in Associations with Cardiometabolic Risk Factors in Term-Born Chinese Children at the Age of 2 Years
Source: Nutrients. 2023 Jul 27;15(15):3342. doi: 10.3390/nu15153342 (PMC10421441; doi:10.3390/nu15153342)
Supplement: Supplementary file 1 [file nutrients-15-03342-s001.zip › nutrients-2470384-supplementary.pdf]

Table S1. The associations between child overweight and obesity and cardio-metabolic risk factors.

	boys				girls			
	n	Mean±SD	β (95% CI)	p value	n	Mean±SD	β (95% CI)	p value
ZWFL	Glucose (mmol/L)				Glucose (mmol/L)			
-1 to 1	148	5.01±0.56	Ref.		138	4.85±0.54	Ref.	
>1 to<=2	43	5.03±0.61	0.01(-0.18,0.20)	0.95	42	4.82±0.69	-0.03(-0.24,0.18)	0.77
>2	9	5.03±0.62	0.01(-0.36,0.39)	0.94	6	5.16±0.35	0.3(-0.19,0.80)	0.23
<-1	15	5.02±0.39	0.01(-0.29,0.30)	0.95	16	5.06±0.93	0.22(-0.10,0.53)	0.18
ZWFL continuous			0.01 (-0.07,0.08)	0.82			-0.00 (-0.10,0.09)	0.94
	Log (insulin), pmol/L				Log (insulin), pmol/L			
-1 to 1	148	3.22±0.91	Ref.		138	3.31±0.82	Ref.	
>1 to<=2	43	3.5±0.84	0.25(-0.06,0.56)	0.11	42	3.46±0.83	0.14(-0.14,0.43)	0.32
>2	9	3.58±1.22	0.34(-0.27,0.95)	0.27	6	3.92±0.77	0.6(-0.07,1.28)	0.08
<-1	15	2.76±0.86	-0.47(-0.94,0.01)	0.06	16	3.19±1	-0.12(-0.55,0.31)	0.58
ZWFL continuous			0.19 (0.07, 0.31)	0.002			0.09 (-0.03, 0.22)	0.14
	total cholesterol (TC), mmol/L				TC, mmol/L			
-1 to 1	148	4.15±0.68	Ref.		138	4.17±0.74	Ref.	
>1 to<=2	43	4.15±0.67	-0.04(-0.27,0.19)	0.74	42	4.35±0.73	0.17(-0.08,0.43)	0.19
>2	9	4.11±0.45	-0.07(-0.52,0.38)	0.76	6	4.46±0.85	0.28(-0.32,0.88)	0.36
<-1	15	3.74±0.89	-0.42(-0.77,-0.06)	0.02	16	4.19±0.83	0.01(-0.37,0.39)	0.95
ZWFL continuous			0.03 (-0.06,0.12)	0.51			0.07 (-0.04,0.18)	0.21
	Triglyceride, mmol/L				Triglyceride, mmol/L			
-1 to 1	148	1.12±0.71	Ref.		138	1.02±0.58	Ref.	
>1 to<=2	43	1.16±0.71	0.02(-0.22,0.25)	0.90	42	1.04±0.42	0.01(-0.17,0.20)	0.90
>2	9	0.93±0.28	-0.21(-0.67,0.25)	0.37	6	1.36±0.65	0.34(-0.10,0.77)	0.14
<-1	15	0.94±0.54	-0.18(-0.54,0.18)	0.34	16	1.26±0.47	0.23(-0.05,0.51)	0.11
ZWFL continuous			0.02 (-0.07,0.12)	0.60			0.01 (-0.07,0.10)	0.73
	HDL, mmol/L				HDL, mmol/L			
-1 to 1	148	1.41±0.34	Ref.		138	1.36±0.29	Ref.	
>1 to<=2	43	1.41±0.38	0(-0.12,0.12)	0.98	42	1.41±0.32	0.05(-0.05,0.15)	0.35
>2	9	1.49±0.46	0.08(-0.16,0.31)	0.51	6	1.26±0.25	-0.11(-0.34,0.13)	0.38
<-1	15	1.44±0.3	0.03(-0.15,0.22)	0.74	16	1.36±0.28	-0.01(-0.16,0.15)	0.94
ZWFL continuous			0.00 (-0.04,0.05)	0.87			0.03 (-0.01,0.08)	0.16
	LDL, mmol/L				LDL, mmol/L			
-1 to 1	148	2.34±0.5	Ref.		138	2.39±0.58	Ref.	
>1 to<=2	43	2.29±0.48	-0.05(-0.22,0.11)	0.52	42	2.48±0.49	0.08(-0.11,0.27)	0.41
>2	9	2.22±0.32	-0.12(-0.45,0.21)	0.48	6	2.57±0.52	0.18(-0.27,0.63)	0.44
<-1	15	2.09±0.52	-0.25(-0.51,0.01)	0.06	16	2.38±0.6	-0.02(-0.31,0.26)	0.88
ZWFL continuous			-0.01 (-0.07,0.06)	0.86			0.03 (-0.05,0.12)	0.41

ZWFL: weight-for-length z-score; All models were adjusted for child age.

Table S2. Age-adjusted partial Pearson correlation coefficients between cardio-metabolic risk factors among term-born Chinese boys and girls aged 2 years

	SBP (mmHg)	DBP (mmHg)	Glucose (mmol/L)	log(insulin), pmol/L	TC (mmol/L)	Triglyceride, mmol/L	HDL, mmol/L
**boys**							
SBP	1						
DBP	0.69***	1					
Glucose	-0.06	-0.04	1				
log(insulin)	0.16*	-0.02	0.43***	1			
TC	-0.04	-0.05	-0.12	0.02	1		
TG	0.04	-0.01	0.11	0.05	0.09	1	
HDL	0.06	0.08	-0.05	0.14	0.41***	-0.21**	1
LDL	-0.12	-0.10	-0.12	-0.02	0.91***	-0.10	0.30***
**girls**	SBP	DBP	Glucose	log(insulin)	TC	TG	HDL
SBP	1						
DBP	0.61***	1					
Glucose	0.003	0.03	1				
log(insulin)	0.09	0.14	0.26**	1			
TC	0.07	-0.01	-0.046	-0.10	1		
TG	0.05	0.11	0.04	0.01	0.001	1	
HDL	0.04	-0.03	-0.08	-0.08	0.41***	-0.32***	1
LDL	0.04	-0.05	-0.03	-0.09	0.94***	-0.10	0.31***

* $p < 0.05$; ** $p < 0.01$; *** $p < 0.001$

TC: total cholesterol

Sample size varies from 558 to 424 in serum measures due to missing blood samples.
